# Weight self-misperception and obesity-related knowledge, attitudes, lifestyle behaviours and cardio-metabolic markers among Chinese school-aged children and adolescents

**DOI:** 10.1017/S1368980023000630

**Published:** 2023-08

**Authors:** Jieyu Liu, Qi Ma, Xinxin Wang, Manman Chen, Tao Ma, Mengjie Cui, Jun Jiang, Yanhui Li, Di Gao, Ying Ma, Wen Yuan, Li Chen, Yi Zhang, Tongjun Guo, Jun Ma, Yanhui Dong

**Affiliations:** 1 Institute of Child and Adolescent Health, School of Public Health, Peking University, National Health Commission Key Laboratory of Reproductive Health, Beijing, 100191, China; 2 School of Public Health and Management, Ningxia Medical University, Key Laboratory of Environmental Factors and Chronic Disease Control, Yinchuan, China; 3 School of Nursing, Peking University, Beijing, China; 4 Department of Obstetrics and Gynecology, Peking University First Hospital, Beijing, China

**Keywords:** Weight perception, Knowledge, Behaviours, Childhood cardio-metabolic markers, China

## Abstract

**Objective:**

The relationships between childhood weight self-misperception and obesity-related factors particularly health markers have not been extensively discussed. This study aims to examine the associations between weight self-misperception and obesity-related knowledge, attitudes, lifestyles and cardio-metabolic markers among Chinese paediatric population.

**Design:**

Cross-sectional study.

**Setting:**

Data sourced from a national survey in Chinese seven provinces in 2013.

**Participants:**

Children and adolescents aged 5–19 years.

**Results:**

Of the total 14 079 participants, there were 14·5 % and 2·2 % participants over-estimated and under-perceived their weight, respectively. Multi-variable logistic regression was applied to calculate OR and 95 % CI (95 % Cl) of obesity-related behaviours and cardio-metabolic markers by actual and perceived weight status. Individuals who perceived themselves as overweight/obese were more likely to have prolonged screen time, insufficient dairy intake and over sugar-sweetened beverages consumption (all *P* < 0·05), regardless of their weight. Furthermore, actual overweight/obese individuals had higher odds of abnormal cardio-metabolic markers, but a smaller magnitude of association was found among weight under-estimators. Among non-overweight/obese individuals, weight over-estimation was positively associated with abdominal obesity (OR: 10·49, 95 % CI: 7·45, 14·76), elevated blood pressure (OR: 1·30, 95 % CI: 1·12, 1·51) and dyslipidemia (OR: 1·43, 95 % CI: 1·29, 1·58).

**Conclusions:**

Weight over-perception was more prevalent than under-estimation, particularly in girls. Weight over-estimators tended to master better knowledge but behave more unhealthily; both weight over-perception and actual overweight/obesity status were associated with poorer cardio-metabolic markers. Future obesity intervention programmes should additionally pay attention to the population with inaccurate estimation of weight who were easily overlooked.

The prevalence of childhood overweight and obesity increased at an alarming rate over the past decades in China^(1)^. Childhood overweight and obesity is correlated with elevated blood pressure (BP)^(2)^ and other chronic disease^(3)^. Apart from the recognised physiological results, depression^(4)^, anxiety^(4)^ and low self-esteem^(5)^ might also result from obesity.

Weight perception refers to an individual’s evaluation of body image with all of the thoughts concerning weight and appearance. Misperception of weight status, defined as under- or over-estimating actual weight, is prevalent among paediatric population^(6)^. It was reported that over one-third of children misperceived their weight status in Guangzhou, China^(7)^. The discrepancy between one’s perceived and actual weight classification may provide insight for healthy behaviours^(7)^. Weight over-perception seemed to be a major contributing factor for the increased prevalence of unhealthy dietary or exercise patterns^(8)^, such as binge eating^(9)^, skipping breakfast and not discussing nutrition topics over meals^(10)^.

While multiple studies have examined the associations between weight misperception and lifestyles, only a few studies have examined the measurable health markers. Sonneville and his colleagues revealed that overweight/obese people who under-perceived their weight gained significantly less weight over time than those who perceive themselves as overweight^(11)^. In addition, overweight/obese adolescent girls who perceived themselves as normal weight had lower BP in adulthood^(12)^. Besides, after adjusting for an extensive range of confounders including socio-demographic profiles, BMI, severe chronic conditions and behaviours (smoking/drinking/exercise), weight status over-perception (*v*. accurate perception) was positively associated with depressive symptoms in women with normal weight^(13)^. Among Korean samples, Kim *et al.* suggested that perception of being obese might be an unfavourable indicator of cardio-metabolic health regardless of actual body weight^(14)^. Among men, those who classified their weight as being obese (*v*. normal) were more likely to have high BP and high TAG, while women tended to develop low HDL-C^(14)^. In the population of Chinese, hypertension and dyslipidemia were negatively associated with weight under-estimation^(15)^, but Mogre et al. reported that participants with higher blood glucose were not significantly associated with under-estimation of weight in Tamale, Ghana^(16)^. Nevertheless, there was a lack of evidence regarding the relationships between weight perception and measurable health markers in Chinese paediatric population. Given the identified associations between weight and multiple cardio-metabolic markers, we hypothesised that weight perception might play an important role in childhood obesity-related health outcomes.

Previous studies investigating the weight perception among Chinese younger-aged populations mainly focused on a single city, such as Guangzhou^(7)^, Wuhan^(17)^, Shandong and Qinghai^(8)^ and Jilin^(18)^. Based on a national sample aged 6–17 years derived from China Health and Nutrition Survey, authors only focused on the effects on dietary intake^(19)^ and dietary weight management behaviours^(20)^, while lack of evidence regarding the impact on negative obesity-related diseases in Chinese paediatric population. To fill this research gap, we sought to examine the associations between weight self-misperception and obesity-related knowledge, attitudes, lifestyle behaviours and multiple cardio-metabolic markers (abdominal obesity, elevated BP, high blood glucose and dyslipidemia) among a nationally representative sample of school-aged children and adolescents, based on cross-sectional data in Chinese seven provinces. We also examined whether the associations differed across sex, age and residence area.

## Methods

### Study population and design

This study used data from a cross-sectional survey of children and adolescents in Chinese seven provinces/cities in 2013, including Hunan, Ningxia, Tianjin, Chongqing, Liaoning, Shanghai and Guangzhou. Briefly, we adopted a multi-stage cluster sampling method to determine the original population. We randomly selected several regions from each province and chose about 12 to 16 primary and secondary schools from each region. Two classes in each grade were randomly selected in these schools. We invited all students and their parents in these selected classes to participate in the survey. Among the original population of 16 637 participants whose physical examination and blood samples were available, 2558 participants were excluded because of missing information on characteristics and weight perception, and the final sample size was 14 079. The project was approved by the Ethical Committee of Peking University (No. IRB0000105213034). Written informed consent was obtained from both students and their parents or legal guardian.

### Data collection and covariates

The child’s questionnaire was performed to collect basic information and lifestyles. Besides, all parents were required to complete structured questionnaire. Both parental and child’s questionnaire of children grades 1–3 were reported by parents. Children from or above the fourth grade would fill in child’s questionnaire by themselves, while instructed by class teachers. After collection, we would recheck the questionnaires by 3 % within one week for the same participants.

Parents were asked to report their own height (cm) and weight (kg), and BMI was calculated as the weight (kg) divided by the square of the height (m^2^). According to the criteria of the Working Group on Obesity in China (WGOC) for Chinese adults^(21)^, BMI cut-offs of 24 and 28 kg/m^2^ were used to classify parental weight status into three categories: ‘normal’, ‘overweight’ and ‘obesity’. Parental educational attainment was grouped into ‘primary school or below’, ‘secondary or equivalent’ and ‘junior college or above’. In addition, we divided residence area into ‘rural’ and ‘urban’ and calculated monthly household income as the sum of monthly income (in CNY, Chinese Yuan) of all household members and classified into < 5000, or ≥ 5000 CNY.

### Self-perception of weight status

Children’s self-perception of weight status was assessed by asking: ‘How do you feel about your current weight status?’, with five response options: ‘very thin’, ‘rather thin’, ‘average’, ‘rather fat’ and ‘very fat’. Those who perceived themselves as ‘very thin’, ‘rather thin’ or ‘average’ were combined into perceived non-overweight/obesity group, as the sample size of these was too small to analyse.

### Anthropometric measurement and group definition

Children’s height (cm) and weight (kg) were measured by trained technicians following a standardised procedure. Height was measured using metal column height-measuring stands (200 cm long with 0·1 cm precision), and weight was measured using lever scales (weights to 120 kg with 0·1 kg precision). BMI (kg/m^2^) was calculated by dividing weight (kg) by height (m) squared. Waist circumference was measured with an accuracy of 0·1 cm using a non-elastic tape at the end of a natural breath at the midpoint between the top of the iliac crest and the lower margin of the last palpable rib. Using a mercury sphygmomanometer (model XJ1ID, China) with the correct cuff size on the right arm of the participant in a relaxed and sitting position, BP was measured. We measured each child twice at 1-min intervals and used the average of the two readings for systolic blood pressure and diastolic blood pressure in final analysis. The stadiometers, lever-type weight scales, non-elastic tape and auscultation mercury sphygmomanometer were all calibrated, and the measuring instruments were similar at all investigated schools.

For students aged 5–17 years old, overweight/obesity was defined as BMI of children ≥ the referent age- and sex-specific 85th percentile according to the reference established by Working Group on Obesity in China^(22)^. For those aged 18–19 years, similar to adults, overweight/obesity was defined as BMI of greater than or equal to 28·0 kg/m^2^. In order to avoid extreme few samples in the group of underweight, we categorised the actual weight status into non-overweight/obesity and overweight/obesity. We combined children’s weight self-perception with actual weight and divided participants into four groups: Group 1: non-overweight/obese participants with accurate estimation; Group 2: weight over-estimators; Group 3: weight under-estimators; Group 4: overweight/obese participants with accurate estimation. Since both the actual weight status and perceived weight status might influence the childhood obesity-related knowledge, attitudes, behaviours or health outcomes, Group 1 was therefore considered as a reference group.

### Definition of abnormal cardio-metabolic markers

Abdominal obesity was defined as waist circumferences ≥ age- and sex-specific 90th percentile^(23)^. Children and adolescents with fasting blood glucose ≥ 5·6 mmol/l were defined as having high blood glucose. Elevated BP referred to systolic blood pressure and/or diastolic blood pressure ≥ 90th percentile for sex, age and height. In this study, the reference values for waist circumference and BP are based on the Chinese population^(23,24)^.

After fasting for 12 h, blood samples were obtained by venipuncture, centrifuged at 3000 rpm for 10 min and then stored at –80°C. All biochemical analyses were carried out at a biomedical analysis company, which was accredited by Peking University^(25)^. Total cholesterol, TAG, LDL-C and HDL cholesterol (HDL-C) were measured by an autoanalyser (TBA-120FR, Toshiba, Tokyo, Japan), with total cholesterol and TAG assayed by enzymatic method, while LDL-C and HDL-C measured using clearance method. Abnormal lipid was defined as follows^(26)^: High total cholesterol referred to total cholesterol ≥ 200 mg/dl. High TAG was considered as TAG ≥ 100 mg/dl in children 9 years old or younger and ≥ 130 mg/dl in adolescents 10 years old and older. High LDL-C was defined as LDL-C ≥ 130 mg/dl, low HDL-C was regarded as HDL-C < 40 mg/dl. A participant with one or more abnormal lipid levels was defined as having dyslipidemia.

### Weight-related knowledge, attitudes and behaviours

Weight-related knowledge was assessed with 10 items. The responses of all items were ‘true’, ‘false’ or ‘don’t know’. Total knowledge score was calculated by summing up the number of items which were correctly answered and deducting points which were incorrectly answered. If the answer was missing or ‘don’t know’, the score of this question was considered to be zero. Attitudes were evaluated with the questions: (1) ‘To what extent do you think obesity is bad for health?’. The responses for question (1) were ‘little’, ‘rather little’, ‘not sure’, ‘rather greatly’ or ‘greatly’. (2) ‘Are you satisfied with your weight status?’ (3) ‘Do you want to change your present weight status?’ and (4) ‘Do you believe you can achieve an ideal weight status through effort?’. The responses were ‘no’, ‘rather no’, ‘not sure’, ‘rather yes’ or ‘yes’ for the questions (2)-(4).

For behaviours, the information of dietary behaviours was collected using questionnaires developed with reference to national Youth Risk Behavior Survey in 2005^(27)^ (Additional information about the national Youth Risk Behavior Survey is available at http://www.cdc.gov/yrbs.). All participants were asked the frequency (days) and servings of vegetables, fruits and sugar-sweetened beverage consumption^(28)^. To better understand the intake of fruit/vegetable, as previous published, one serving was defined as the size of an ordinary adult’s closed fist and roughly equalled a medium-sized apple or orange (≈200 g)^(29)^. One serving of sugar-sweetened beverage was determined as a canned beverage (approximately 250 ml)^(30)^. The daily dietary intake of single food was calculated as follows: average daily intake = (days of consumption × servings in those days)/7. A daily consumption of 2∼3 servings (300∼500 g) of vegetables^(31)^ and 2 servings (400 g) of fruits^(32)^ is recommended for children and adolescents in China; insufficient vegetable and fruit consumption was therefore defined as < 3 servings/d and < 2 servings/d, respectively. In addition, based on Chinese dietary guidelines for children^(33)^, we defined over sugar-sweetened beverage consumption as > 1 servings/week. Children also reported frequency (days or times) of breakfast, dairy products and fried food (e.g. fried chicken and fried potatoes) intake over the preceding 7 d. Dairy products included low-/full-fat milk, cheese, yogurt and cottage cheese, except for the mixed foods that contain milk such as butter and ice cream. Participants were asked ‘How many days, over the past 7 d, have you consumed dairy products?’. Indeed, data on individual dairy products were not available. According to the dietary guidelines for school-age children in China (2016)^(33)^, breakfast skipping and insufficient dairy intake were considered as < 7 d/week, excessive fried food consumption was defined as > 1 d/week.

For lifestyle behaviours, we recorded child’s physical activity using the Chinese version of International Physical Activity Questionnaire-Short Form^(34)^, that is always a useful instrument for generating internationally comparable data on in Chinese population^(35)^. Moderate-to-vigorous physical activity was defined as any kind of aerobic activity that increased heart rate and breathing, such as running, basketball, football, swimming. Moderate-to-vigorous physical activity was asked by the following questions: ‘How many days, over the past 7 d, have you done moderate-to-vigorous physical activity? And how much time did you last on average?’ Children reported the frequency (days) and duration (hours and minutes) for moderate-to-vigorous physical activity over the past 7 d, and the average daily time was calculated as follows: (days × duration in each of those days)/7. We defined physical inactivity as moderate-to-vigorous physical activity < 1 h/d. The questionnaires collecting other lifestyle behaviours were developed based on the Information, Motivation and Behavioural skills Model^(36)^. Sitting and screen time were asked by the following question: ‘Over the past 7 d, how much time did you spend on sitting or lying down at school and at home (not including sleeping)/watching TV or playing computer or video games on average?’, students reported the duration (hours and minutes) of sitting and screening per day, and both the prolonged sitting time and screen time were defined as ≥ 2 h/d.

Unhealthy lifestyle score was calculated as follows: if one of the above-mentioned unhealthy lifestyles was satisfied, one point was added; otherwise, one point was deducted.

### Statistical analysis

Continuous and categorical variables were presented as mean ± sd and frequency (percentage), respectively. Differences in demographic characteristics by four groups were examined by one-way ANOVA test for continuous variables and Pearson’s chi-squared test for categorical variables. Kappa test was used to evaluate the agreement between the perceived weight and actual weight status, with values of 0·00 to 0·20 considered slight, 0·21 to 0·40 fair, 0·41 to 0·60 moderate, 0·61 to 0·80 substantial and 0·81 to 1·00 almost perfect agreement. We also adopted one-way ANOVA and Pearson’s chi-squared test to examine the difference between the four groups, with Bonferroni-corrected post hoc tests for multiple comparisons. The significance of Bonferroni formula was set at *α* = 0·0125. Meanwhile, multivariate logistic regression model was applied to calculate OR (95 % confidence level (95 % CI)) of obesity-related behaviours and abnormal cardio-metabolic markers by actual and perceived weight status. Potential confounders were adjusted in the multivariate logistic regression model, including age, sex, residence area, ethnicity, family incomes, parental educational attainment and parental weight status. Furthermore, stratified analyses were performed to explore the differences according to sex (boys, girls), age (5–9 years old, 10–14 years old, 15–19 years old) and residence area (rural, urban). Data were not shown if the number of participants in this group was too small to analyse. All statistical analyses were performed using Statistical Analysis System (SAS) software (version 9.4, SAS Institute), and we deemed *P*-values less than 0·05 to be statistically significant.

## Results

### Characteristic of participants

Of the 14 079 children, there were 2035 (14·5 %) weight over-estimators and 309 (2·2 %) under-estimators (Table 1). In general, weight over-estimators (Mean age = 12·4 years, sd = 3·0) tended to be older than those under-estimators (Mean age = 9·2 years, sd = 2·9) and accurate-estimators. The BMI values (kg/m^2^) of children aged 5–9 years increased from 15·6 of non-overweight/obese children with accurate estimation to 18·1 of weight over-estimators. Similar escalating trend of BMI was observed in children aged 10–14 years and 15–19 years (*P* < 0·01). In addition, most of individuals’ parents in four groups attained education of secondary or equivalent, and most of the participants who over-perceived as overweight/obesity came from urban areas, while those who under-perceived their weight mostly came from rural areas. The multiple comparisons of baseline information between groups were described in Supplementary Table 1.

**Table 1:** Baseline characteristic of included population

Characteristics	Actual weight: non-overweight/obesity				Actual weight: overweight/obesity				*P*-value*
	Group 1: perceived non-overweight/obesity (*n* 9996)		Group 2: perceived overweight/obesity (*n* 2035)		Group 3: perceived non-overweight/obesity (*n* 309)		Group 4: perceived overweight/obesity (*n* 1739)		
	Mean	sd	Mean	sd	Mean	sd	Mean	sd	
Age, year	10·9	3·3	12·4	3·0	9·2	2·9	11·4	3·3	< 0·01
BMI values									
5–9 years old	15·6	1·6	18·1	1·8	21·1	3·4	22·6	2·9	< 0·01
10–14 years old	17·6	2·0	20·4	1·8	24·4	3·2	26·0	2·9	< 0·01
15–19 years old	19·3	1·9	21·9	2·0	26·5	2·4	28·1	3·1	< 0·01
	*n*	%	*n*	%	*n*	%	*n*	%	
Sex									< 0·01
Boys	5135	51·37	892	43·83	134	43·37	898	51·64	
Girls	4861	48·63	1143	56·17	175	56·63	841	48·36	
Residence area									< 0·01
Urban	5154	51·6	1147	56·4	157	50·8	1013	58·3	
Rural	4842	48·4	888	43·6	152	49·2	726	41·8	
Ethnicity									0·005
Han	9248	92·5	1875	92·1	295	95·5	1623	93·3	
Hui	354	3·5	83	4·1	5	1·6	41	2·4	
Tibetan	20	0·2	8	0·4	0	0·0	3	0·2	
Mongolian	145	1·5	17	0·8	0	0·0	21	1·2	
Other	229	2·3	52	2·6	9	2·9	51	2·9	
Paternal weight status									< 0·01
Normal	6062	60·6	1108	54·5	133	43·0	703	40·4	
Overweight	3122	31·2	679	33·4	119	38·5	697	40·1	
Obesity	812	8·1	248	12·2	57	18·5	339	19·5	
Maternal weight status									< 0·01
Normal	8246	82·5	1540	75·7	220	71·2	1166	67·1	
Overweight	1462	14·6	403	19·8	72	23·3	427	24·6	
Obesity	288	2·9	92	4·5	17	5·5	146	8·4	
Paternal educational attainment									0·495
Primary school or below	665	6·7	126	6·2	18	5·8	96	5·5	
Secondary or equivalent	5838	58·4	1183	58·1	189	61·2	1046	60·2	
Junior college or above	3493	34·9	726	35·7	102	33·0	597	34·3	
Maternal educational attainment									0·434
Primary school or below	884	8·8	175	8·6	28	9·1	133	7·7	
Secondary or equivalent	5740	57·4	1148	56·4	188	60·8	1016	58·4	
Junior college or above	3372	33·7	712	35·0	93	30·1	590	33·9	
Monthly household income									0·047
< 5000 CNY	8425	84·3	1729	85·0	252	81·6	1428	82·1	
≥ 50 000 CNY	1571	15·7	306	15·0	57	18·5	311	17·9	

Abbreviation: CNY, Chinese Yuan.

* Differences of items between children and adolescents in four groups were evaluated using one-way ANOVA and Pearson’s chi-square tests.

### Consistency between perception of weight and actual weight status

Supplementary Table 2 presented the Kappa results showing the consistency between children’s self-perception of weight and actual weight status. Of included children and adolescents, whilst there was a high degree of accuracy of weight status perception in those of non-overweight/obesity weight (70·8 %), there was also a considerable proportion of individuals over-estimated their weight, approximately accounting for 14·5 %. The consistency in the total population was of moderate agreement (Kappa = 0·504, *P* < 0·01). Similar moderate agreements were observed for boys (Kappa = 0·554, *P* < 0·01) and girls (Kappa = 0·457, *P* < 0·01), but such weight over-estimation in those of non-overweight/obesity mainly occurred in girls than boys (girls: 16·3 % *v.* boys: 12·6 %). Similar high rates of weight over-estimation were also detected among 15- to 19-year-age individuals and those with urban residence.

### Prevalence of abnormal cardio-metabolic markers by actual and perceived weight status

Of the 14 079 children and adolescents, most of the prevalence of abnormal cardio-metabolic markers, except for high blood glucose, differed by the four groups (*P* < 0·01) (Table 2). Consistent with traditional cognition, individuals whose actual weight was overweight/obesity had higher prevalence of negative health outcomes. Besides, in the non-overweight/obesity groups, weight over-estimators also had higher prevalence of abnormal cardio-metabolic markers (high BP: 33·7 %; dyslipidemia: 35·2 %) than their counterparts who had accurate awareness of weight status (high BP: 24·1 %; dyslipidemia: 27·0 %). Age- and residence-specific prevalence of abnormal cardio-metabolic markers showed similar trends (online Supplementary Table 3).

**Table 2:** Prevalence of abnormal cardio-metabolic markers by self-perception combined with actual weight status

Cardio-metabolic markers, *n* (%)	Actual weight: non-overweight/obesity				Actual weight: overweight/obesity				*P*-value	Significant multiple group comparisons*
	Group 1: perceived non-overweight/obesity		Group 2: perceived overweight/obesity		Group 3: perceived non-overweight/obesity		Group 4: perceived overweight/obesity			
	*n*	%	*n*	%	*n*	%	*n*	%		
Total population	(*n* 9996)		(*n* 2035)		(*n* 309)		(*n* 1739)			
Abdominal obesity	52	0·5	100	4·9	113	36·6	1135	65·3	< 0·01	Group 4 > 3 > 2 > 1
Elevated BP	2412	24·1	685	33·7	104	33·7	795	45·7	< 0·01	Group 4 > 3 > 1; Group 4 > 2 > 1;
High blood glucose	170	1·7	34	1·7	9	2·9	45	2·6	0·032	Group 3 > 2, Group 3 > 1
Dyslipidemia	2701	27·0	716	35·2	125	40·5	861	49·5	< 0·01	Group 3 > 1; Group 4 > 2 > 1;
High TC	486	4·9	116	5·7	20	6·5	145	8·3	< 0·01	Group 4 > 2; Group 4 > 1
High TAG	1695	17·0	457	22·5	92	29·8	613	35·3	< 0·01	Group 4 > 2 > 1; Group 3 > 1
High LDL-C	243	2·4	73	3·6	12	3·9	100	5·8	< 0·01	Group 4 > 2; Group 4 > 1
Low HDL-C	994	9·9	330	16·2	49	15·9	433	24·9	< 0·01	Group 4 > 3 > 1; Group 2 > 1;
Boys	(*n* 5135)		(*n* 892)		(*n* 134)		(*n* 898)			
Abdominal obesity	18	0·4	35	3·9	55	41·0	598	66·6	< 0·01	Group 4 > 3 > 2 > 1
Elevated BP	1364	26·6	327	36·7	48	35·8	450	50·1	< 0·01	Group 4 > 2 > 1; Group 4 > 3 > 1
High blood glucose	124	2·4	21	2·4	6	4·5	31	3·5	0·146	NS
Dyslipidemia	1289	25·1	321	36·0	49	36·6	461	51·3	< 0·01	Group 4 > 2 > 1; Group 3 > 1
High TC	203	4·0	57	6·4	14	10·5	74	8·2	< 0·01	Group 4 > 1; Group 3 > 1; Group 2 > 1
High TAG	774	15·1	176	19·7	34	25·4	321	35·7	< 0·01	Group 4 > 2 > 1; Group 3 > 1
High LDL-C	98	1·9	37	4·2	6	4·5	53	5·9	< 0·01	Group 4 > 1; Group 3 > 1
Low HDL-C	553	10·8	165	18·5	19	14·2	239	26·6	< 0·01	Group 4 > 2 > 1; Group 4 > 3
Girls	(*n* 4861)		(*n* 1143)		(*n* 175)		(*n* 841)			
Abdominal obesity	34	0·70	65	5·7	58	33·1	537	63·9	< 0·01	Group 4 > 3 > 2 > 1
Elevated BP	1048	21·6	358	31·3	56	32·0	345	41·0	< 0·01	Group 4 > 2 > 1, Group 3 > 1
High blood glucose	46	1·0	13	1·1	3	1·7	14	1·7	0·242	NS
Dyslipidemia	1412	29·1	395	34·6	76	43·4	400	47·6	< 0·01	Group 4 > 2 > 1; Group 3 > 1
High TC	283	5·8	59	5·2	6	3·4	71	8·4	0·005	Group 4 > 3
High TAG	921	19·0	281	24·6	58	33·1	292	34·7	< 0·01	Group 4 > 1; Group 3 > 1
High LDL-C	145	3·0	36	3·2	6	3·4	47	5·6	0·002	Group 4 > 1
Low HDL-C	441	9·1	165	14·4	30	17·1	194	23·1	< 0·01	Group 4 > 2 > 1; Group 3 > 1

BP, blood pressure; TC, total cholesterol; TAG, triglyceride

* Chi-squared test with Holm Bonferroni correction was used to account for multiple comparisons. NS, not significant.

### Obesity-related knowledge, attitude and behaviours by actual and perceived weight status

Table 3 presented obesity-related knowledge by weight estimation in two BMI categories. The correct rates of most knowledge items did not differ between four groups. However, for some items (e.g. meat and western fast food), children and adolescents who estimated themselves as overweight/obesity had higher correct rates than their counterparts, regardless of their actual weight status (*P* < 0·05). In total, non-overweight/obese children’s over-estimation was associated with the highest knowledge scores of 9·68 (sd = 2·3), while the overweight/obese children and adolescents who under-estimated their weight status had the lowest correct rates of 9·48 (sd = 2·5). Girls and adolescents aged 15–19 years old were better at mastering weight-related knowledge.

**Table 3:** Children’s weight-related knowledge by actual and perceived weight status

Knowledge items/scores	Actual weight: non-overweight/obesity				Actual weight: overweight/obesity				Significant multiple group comparisons*
	Group 1: perceived non-overweight/obesity		Group 2: perceived overweight/obesity		Group 3: perceived non-overweight/obesity		Group 4: perceived overweight/obesity		
	*n*	%	*n*	%	*n*	%	*n*	%	
Knowledge items	(*n* 9996)		(*n* 2035)		(*n* 309)		(*n* 1739)		
It is harmful to watch TV, play computer games and surf the internet for a long time. (yes)	9208	92·4	1874	92·3	278	90·6	1589	91·6	NS
It is not necessary to exercise every day. (no)	8929	89·8	1861	91·6	282	92·2	1567	90·4	NS
Fruits and vegetables should be eaten every day. (yes)	9756	97·8	1991	98·1	301	97·4	1706	98·3	NS
Meat contains fat and protein and the more you eat meat, the better. (no)	8983	90·2	1926	94·8	285	92·8	1631	94·0	Group 2 > 1; Group 4 > 1
It is healthier to drink plain boiled water than sugar-sweetened beverages. (yes)	8480	85·1	1735	85·4	250	81·2	1480	85·3	NS
You don’t have to eat breakfast as long as you eat more lunch. (no)	9708	97·4	1986	97·7	297	96·1	1683	97·0	NS
It is harmful to eat too much fried food. (yes)	9216	92·5	1893	93·2	278	90·3	1620	93·3	NS
Western fast food (i.e. KFC, McDonald’s, etc) is more nutritious. (no)	9335	93·7	1939	95·4	288	93·2	1645	94·8	Group 2 > 1
It is beneficial to your health to drink milk every day. (yes)	9523	95·6	1915	94·3	291	94·5	1636	94·4	NS
It is beneficial to your health to eat more high-energy snacks. (no)	9501	95·3	1958	96·4	295	95·5	1678	96·7	NS
Often eating out is not good for your health. (yes)	8343	83·8	1675	82·8	257	83·2	1454	84·1	NS
Of the ‘Chinese Residents’ Balanced Diet Pagoda’, the food at the bottom should be eaten more. (no)	3468	35·3	770	38·3	124	41·2	651	38·0	NS
Knowledge scores	Mean	sd	Mean	sd	Mean	sd	Mean	sd	
Total	9·50	2·5	9·68	2·3	9·48	2·5	9·64	2·4	NS
Boys	9·40	2·6	9·43	2·5	9·35	2·8	9·59	2·5	NS
Girls	9·62	2·4	9·87	2·1	9·57	2·3	9·69	2·4	Group 2 > 1
5–9 years old	9·37	2·6	9·39	2·6	9·42	2·6	9·48	2·7	NS
10–14 years old	9·48	2·5	9·63	2·3	9·40	2·5	9·65	2·4	NS
15–19 years old	9·83	2·2	9·92	2·2	10·22	1·7	9·84	2·2	NS
Rural area	9·46	2·6	9·73	2·2	9·57	2·6	9·75	2·4	NS
Urban area	9·55	2·4	9·64	2·4	9·39	2·4	9·56	2·5	NS

* One-way ANOVA and chi-squared test with Holm Bonferroni correction were used to account for multiple comparisons. NS, not significant.

Table 4 showed obesity-related attitudes by weight estimation in BMI categories. Participants who perceived themselves to be overweight/obese were more likely to be unsatisfied with their weight (71·5 % for non-overweight/obese participants and 84·0 % for overweight/obese participants) and had higher intention to change their present weight status (84·2 % for non-overweight/obese participants and 90·2 % for overweight/obese participants), especially for girls. In subgroup analysis (online Supplementary Table 4), 15- to 19-year-aged adolescents showed higher unsatisfactory with their present weight.

**Table 4:** Children’s weight-related attitudes by actual and perceived weight status

Weight-related attitudes, *n* (%)	Actual weight: non-overweight/obesity				Actual weight: overweight/obesity				Significant multiple group comparisons*
	Group 1: perceived non-overweight/obesity		Group 2: perceived overweight/obesity		Group 3: perceived non-overweight/obesity		Group 4: perceived overweight/obesity		
	*n*	%	*n*	%	*n*	%	*n*	%	
Total population	(*n* 9996)		(*n* 2035)		(*n* 309)		(*n* 1739)		
To what extent do you think obesity is bad for health? (greatly or rather greatly)	7751	77·5	1637	80·4	247	80·0	1402	80·6	Group 2 > 1, Group 4 > 1
Are you satisfied with your weight status? (no or rather no)	2957	29·6	1455	71·5	91	29·5	1461	84·0	Group 4 > 2 > 3, Group 4 > 2 > 1
Do you want to change your present weight status? (yes or rather yes)	4192	41·9	1713	84·2	169	54·7	1569	90·2	Group 4 > 2 > 3 > 1
Do you believe you can achieve an ideal weight status through effort? (yes or rather yes)	6622	66·3	1394	68·5	220	71·2	1226	70·5	Group 3 > 1
Boys	(*n* 5135)		(*n* 892)		(*n* 134)		(*n* 898)		
To what extent do you think obesity is bad for health? (greatly or rather greatly)	3897	75·9	692	77·6	114	85·1	744	82·9	Group 3 > 1
Are you satisfied with your weight status? (no or rather no)	1518	29·6	528	59·2	35	26·1	752	83·8	Group 4 > 2 > 1, Group 4 > 2 > 3
Do you want to change your present weight status? (yes or rather yes)	2057	40·1	685	76·8	63	47·0	824	91·9	Group 4 > 2 > 1, Group 4 > 2 > 3
Do you believe you can achieve an ideal weight status through effort? (yes or rather yes)	3371	65·6	622	69·7	97	72·4	662	73·8	Group 4 > 1
***Girls***	(*n* 4861)		(*n* 1143)		(*n* 175)		(*n* 841)		
To what extent do you think obesity is bad for health? (greatly or rather greatly)	3839	79·0	914	80·0	148	84·6	689	81·9	NS
Are you satisfied with your weight status? (no or rather no)	1432	29·5	890	77·9	63	36·0	746	88·7	Group 4 > 2 > 1, Group 4 > 2 > 3
Do you want to change your present weight status? (yes or rather yes)	2124	43·7	991	86·7	117	66·9	782	93·0	Group 4 > 3 > 1, Group 2 > 1
Do you believe you can achieve an ideal weight status through effort? (yes or rather yes)	3237	66·6	743	65·0	137	78·3	593	70·5	NS

* Chi-squared test with Holm Bonferroni correction was used to account for multiple comparisons. NS, not significant.

Table 5 showed weight-related behaviours by weight perception in two BMI categories. Overall, children and adolescents who perceived to be overweight/obese, no matter they were overweight/obesity or not, were more likely to behave unhealthily, such as having prolonged screen time and unhealthy diet (e.g. insufficient dairy intake and over sugar-sweetened beverage intake), compared to the reference group. On the contrary, the weight under-estimators (Group 3) tended to live healthier life such as more physical activities doing and fruit consumption (*P* < 0·05). The total unhealthy lifestyle scores were the highest among weight over-estimators with normal weight/underweight. Specifically, girls and children aged 5–9 years and with urban residence tended to behave healthily.

**Table 5:** Multivariate OR and 95 % CI for unhealthy lifestyle by groups of self-perception combined with actual weight status

Unhealthy Lifestyle	Actual weight: non-overweight/obesity				Actual weight: overweight/obesity				Significant multiple group comparisons‡
			Group 2: perceived overweight/obesity		Group 3: perceived non-overweight/obesity		Group 4: perceived overweight/obesity		
	Group 1: perceived non-overweight/obesity		OR	95 % CI	OR	95 % CI	OR	95 % CI	
Model†	(*n* 9996)		(*n* 2035)		(*n* 309)		(*n* 1739)		
Physical inactivity	1 (Reference)		1·10	0·95, 1·27	0·73	0·55, 0·98**	1·05	0·90, 1·22	
Prolonged screen time	1 (Reference)		1·13	1·01, 1·26*	0·98	0·75, 1·29	1·20	1·07, 1·35**	
Prolonged sitting time	1 (Reference)		1·09	0·96, 1·25	0·87	0·66, 1·15	0·99	0·86, 1·14	
Insufficient sleep duration	1 (Reference)		1·11	0·98, 1·26	1·38	0·97, 1·96	0·97	0·84, 1·12	
Breakfast skipping	1 (Reference)		1·35	1·20, 1·52**	0·88	0·61, 1·27	1·11	0·96, 1·27	
Insufficient dairy intake	1 (Reference)		1·14	1·03, 1·27**	0·98	0·78, 1·24	1·16	1·04, 1·29**	
Excessive fried food	1 (Reference)		0·97	0·88, 1·07	1·25	0·99, 1·59	0·98	0·88, 1·09	
Insufficient vegetable intake	1 (Reference)		1·05	0·93, 1·19	0·96	0·72, 1·28	0·95	0·83, 1·08	
Insufficient fruit intake	1 (Reference)		1·21	1·08, 1·36**	0·76	0·60, 0·97**	0·96	0·85, 1·08	
Over sugar-sweetened beverages	1 (Reference)		1·13	1·01, 1·26*	0·85	0·63, 1·16	1·21	1·07, 1·36**	
Unhealthy lifestyle scores	Mean	sd	Mean	sd	Mean	sd	Mean	sd	
Total**	1·7	3·4	2·6	3·5	0·9	3·0	2·0	3·5	Group 2 > 1 > 3, Group 4 > 3
Boys**	2·0	3·4	2·6	3·7	1·0	2·8	2·3	3·5	Group 2 > 3, Group 4 > 3
Girls**	1·4	3·3	2·6	3·3	0·8	3·1	1·8	3·3	Group 2 > 1 > 3, Group 4 > 3
5–9 years old**	0·9	3·0	1·5	3·1	0·8	2·9	1·3	3·2	Group 2 > 3
10–14 years old**	1·7	3·4	2·2	3·5	0·8	3·0	1·9	3·6	Group 2 > 3, Group 4 > 3
15–19 years old	3·3	3·3	3·7	3·3	2·4	3·2	3·3	3·2	NS
Rural area	1·8	3·3	2·7	3·3	0·9	2·7	2·2	3·5	NS
Urban area	1·6	3·4	2·4	3·6	0·9	3·2	1·9	3·4	NS

*P < 0·05, **P < 0·01.

† Model: adjusted for sex, age, residence area, ethnicity, incomes, parental educational attainment, parental weight.

‡ One-way ANOVA with Holm Bonferroni correction was used to account for multiple comparisons. NS, not significant.

### Cardio-metabolic markers by actual and perceived weight status

**Fig. 1:**
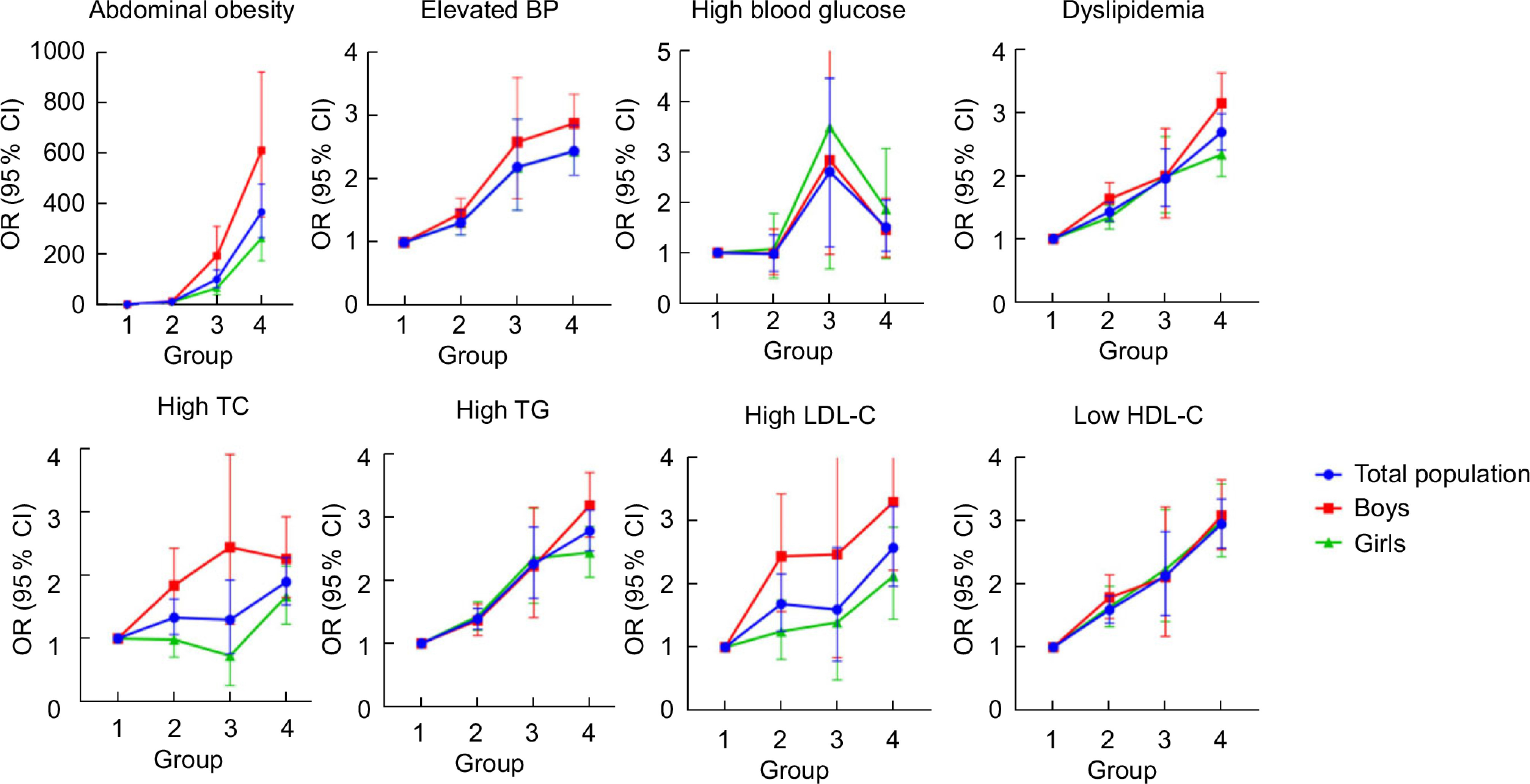
Multivariate OR and 95 % CI for abnormal cardio-metabolic markers by groups of self-perception combined with actual weight status, stratified by sex. (Group 1: non-overweight/obese participants with accurate estimation; Group 2: weight over-estimators; Group 3: weight under-estimators; Group 4: overweight/obese participants with accurate estimation. Group 1 was considered as a reference group; BP, blood pressure; TC, total cholesterol; TAG, triglyceride. 95 %CI did not contain 1 referred to *P* < 0·05.)

The OR for cardio-metabolic markers showed escalating trend among the four groups combining weight self-perception with actual weight status, especially for abdominal obesity, elevated BP and dyslipidemia (Fig. 1 and online Supplementary Table 5). Consistent with conventional knowledge, the OR of abnormal cardio-metabolic markers were more pronounced among overweight/obese individuals than non-overweight/obese groups, but smaller magnitudes of these associations were found among weight under-estimators with overweight/obesity, that was, though they were not aware of their overweight status, they still faced high likelihoods of abnormal cardio-metabolic markers (*P* < 0·05). Notably, both perception of overweight/obesity and actual overweight/obesity status were associated with greater odds of adverse health outcomes, and the two had joint associations. Apart from the traditional target of overweight/obese individuals with accurate estimation, those weight over-estimators with normal weight and underweight were also at higher odds of abdominal obesity (OR: 10·49, 95 % CI: 7·45, 14·76), elevated BP (OR: 1·30, 95 % CI: 1·12, 1·51) and dyslipidemia (OR: 1·43, 95 % CI: 1·29, 1·58), compared with those non-overweight/obese individuals with accurate estimation. Considering the sex differences, the OR for abnormal cardio-metabolic markers in relation to the weight misperception seemed to be much larger among boys than girls.

Similar escalating OR of abnormal cardio-metabolic markers in these four groups were detected among various subgroups divided by age and residence areas, but large OR of abdominal obesity, elevated BP and dyslipidemia were more evident among children and adolescents aged 15–19 years old (*P* < 0·05), and those came from rural area were more likely to have higher likelihoods of abdominal obesity and dyslipidemia associated with body weight misperception (P < 0·05) (online Supplementary Tables 6, 7 and Supplementary Fig. 1, 2).

## Discussion

To our knowledge, there were 16·6 % participants who misperceived their weight and the consistency between the perceived weight and actual weight was only of moderate agreement. Weight over-estimators tended to master better knowledge but behave more unhealthily, while weight under-estimators exactly the opposite. Both weight over-perception and actual overweight/obesity status were associated with greater odds of adverse health outcomes. That was, individuals who estimated them to be overweight/obese already faced potentially higher likelihoods of cardio-metabolic risks, even among those with normal weight.

Previous findings found that under-estimation of weight was much more prevalent than over-estimation among children in southern China^(7)^; however, we concluded that over-estimation of weight was more prevalent than under-estimation across China, particularly among girls, adolescents aged 15–19 years old and individuals came from urban area. The only moderate agreement between the perceived weight and actual weight was not surprising, as girls tended to over-estimate their weight and selected a thinner ideal body shape^(37)^, and adolescents aged older might be more sensitive about weight than younger counterparts. The above phenomenon was also applicable to students living in urban area, without the trouble and burden of other living aspects; they might be more conscious about their weight and body shape.

Findings indicated that individuals who perceived themselves as being overweight or obese were better at mastering obesity-related knowledge and had higher intention to lose weight but were more likely to adopt unhealthy lifestyles. Prior research also showed that weight over-estimators were more likely to adopt unhealthy behaviours^(19)^ and were less physically active^(38)^. Most of participants who recognised their overweight/obesity status had higher intention to change weight because they realised that it was expected of them, but weight loss was not an intrinsic goal for them. For this reason, the self-estimation of weight could only influence their satisfactions but not their behaviours^(39)^. The results of subgroups should be considered while tailoring suitable obesity intervention strategies. Specifically, female Chinese adolescents would prefer a thinner body figure as their ideal body image^(37)^; thus, girls might have higher prevalence of body image concerns, higher scores of knowledge and more healthy lifestyles. Compared to younger children, adolescents were better at mastering obesity-related knowledge, as they might obtain more education in school. However, they tended to adopt unhealthy behaviours since they lived in schools and had freedom to lead their favourable lifestyles. In addition, we have to acknowledge that the economic transitions in China have been linked to shifts in beauty ideals in individuals living in metropolitan or rural areas, and the attitudes towards one’s body are influenced by dominant culture. Since individuals living in urban area tended to be more conscious about their body shape, they would select healthy behaviours. Unfortunately, the current national survey could not distinguish the cultural differences, which prevent us to further investigate the impacts of culture patterns.

The evidence that misperception of weight was associated with unhealthy lifestyles raised the question of whether this misperception was associated with obesity-related markers. In the present study, those participants who over-perceived their weight were also at high odds of deleterious health outcomes. In addition to the unhealthy habits, we speculated that individuals with overweight/obesity are subject to discrimination, teasing and negative comments from peers^(40)^, leading to increased stress and other psychological disorders^(41)^. Data from UK birth cohorts found that the magnitude of association between overweight perception and depressive symptoms in girls had increased over the past 30 years^(42)^. Although the mechanism was not fully understood, such psycho-social stress could increase the likelihoods of elevated BP^(43)^ and diabetes^(44)^. Weight-based self and social stigmatisation was also a powerful risk factor for incident obesity^(45)^.

Early identification of children’s weight misperception along with healthy lifestyle promotion shaped a crucial role in abnormal obesity-related cardio-metabolic markers confrontation; therefore, public health campaigns should address weight stigma and avoid the use of body dissatisfaction as a motivator for weight change. Since the perception of overweight/obesity and actual overweight/obesity status were both positively associated with adverse health outcomes, and in line with the notion that ‘perception’ was more powerful than ‘reality’ in terms of weight^(46)^, the traditional high-risk population with class III obesity should be updated. Schools and parents should take targeted measures to help children and adolescents increase their accurate awareness of weight. Apart from this, increasing efforts should consider strategies to reduce weight stigma as part of broader initiatives, such as providing stigma-reduction training for health care professionals. Sex, age and residence area differences should also be paid attention while tailoring suitable intervention programmes.

Strengths of this study included the large sample size and nationally multi-centre and representative population. However, several limitations should be noted. Firstly, our results may not be applicable to other ethnic groups since most of the study population was of Han ethnicity. Secondly, BMI may over-estimate fatness in children who were shorter and may under-estimate adiposity in those with reduced muscle mass^(47)^. Since body composition could better predict body size dissatisfaction^(48)^, further investigation of weight categories should be based on the body composition. Thirdly, the self-reported lifestyle behaviours could lead to a certain degree of recall bias. On this process, we carried out strict quality control to ensure the reliability. In addition, the behaviour data recall of 7 d might represent the recent lifestyle habits; a pilot study using photo tracking could be used to accurately assess the dietary habits. Also, in order to fill the questionnaire in a minimum time, we could not rule out that excessive junk food (pastries, chocolates, candies, etc.) or insufficient protein foods, might also affect cardio-metabolic markers. In addition, parental weight and height were self-reported, but the self-reported data of Chinese adults had fairly good agreement with each other. For reasons of cost and practicality, self-reported data are still often used in epidemiological surveillance^(49)^. Fourthly, this study did not consider psychological factors which had been shown to influence weight perception; future studies stratified by personal psychological levels are needed. Apart from this, the whole-body silhouettes should be used to assess perceived body habitus. Fifth, culture preferences play a crucial role in body size estimation, but due to difficulties in the measurement of acculturation, the comparison remains problematic. Besides, the International Physical Activity Questionnaire-Short Form questionnaire for physical activity was not completely suitable for children. However, the open-ended questions contained in International Physical Activity Questionnaire-Short Form can represent the levels of childhood physical activities to some extent. Finally, the nature of cross-sectional study design may be less effective to investigate the direction of causal relationship.

In conclusion, weight over-estimators tended to master better knowledge but behave more unhealthily, while weight under-estimators exactly the opposite. Both weight over-perception and actual overweight/obesity status were positively associated with abdominal obesity, elevated BP and dyslipidemia. Future national obesity-related intervention programmes should additionally pay attention to the population with inaccurate estimation of body weight.

## Supplementary material

For supplementary material accompanying this paper visit https://doi.org/10.1017/S1368980023000630. click here to view supplementary material
