# Supplementary material for: Weight self-misperception and obesity-related knowledge, attitudes, lifestyle behaviours and cardio-metabolic markers among Chinese school-aged children and adolescents
Source: Public Health Nutr. 2023 Apr 24;26(8):1549–61. doi: 10.1017/S1368980023000630 (PMC10410384; doi:10.1017/S1368980023000630)
Supplement: Supplementary file 1 [file S1368980023000630sup001.docx]

**Weight self-misperception and Obesity-related Knowledge, Attitudes, Lifestyle Behaviors and Cardio-metabolic Markers among Chinese School-aged Children and Adolescents**

**Supplementary File**

**Supplementary Table 1.** Significant multiple group comparisons of baseline characteristic of included population.

**Supplementary Table 2.** Consistency analysis of perception of child's weight and actual weight status.

**Supplementary Table 3.** Prevalence of abnormal cardio-metabolic markers by self-perception combined with actual weight status in sub-groups.

**Supplementary Table 4.** Children's weight-related attitudes by self-perception combined with actual weight status in sub-groups.

**Supplementary Table 5.** Multivariate odds ratios (OR) and 95% confidence intervals (CI) for abnormal cardio-metabolic markers by groups of self-perception combined with actual weight status, stratified by sex.

**Supplementary Table 6.** Multivariate odds ratios (OR) and 95% confidence intervals (CI) for abnormal cardio-metabolic markers by groups of self-perception combined with actual weight status, stratified by age.

**Supplementary Table 7.** Multivariate odds ratios (OR) and 95% confidence intervals (CI) for abnormal cardio-metabolic markers by groups of self-perception combined with actual weight status, stratified by residence areas.

**Supplementary Figure 1.** Multivariate odds ratios (OR) and 95% confidence intervals (CI) for abnormal cardio-metabolic markers by groups of self-perception combined with actual weight status, stratified by age. (Group 1: non-overweight/obese participants with accurate estimation; Group 2: weight over-estimators; Group 3: weight under-estimators; Group 4: overweight/obese participants with accurate estimation. Group 1 was considered as a reference group; BP, blood pressure; TC, total cholesterol; TG, triglyceride; LDL-C, low-density lipoprotein cholesterol; HDL-C, high-density lipoprotein cholesterol. 95%CI did not contain 1 referred to *P*<0.05.)

**Supplementary Figure 2.** Multivariate odds ratios (OR) and 95% confidence intervals (CI) for abnormal cardio-metabolic markers by groups of self-perception combined with actual weight status, stratified by residence areas. (Group 1: non-overweight/obese participants with accurate estimation; Group 2: weight over-estimators; Group 3: weight under-estimators; Group 4: overweight/obese participants with accurate estimation. Group 1 was considered as a reference group; BP, blood pressure; TC, total cholesterol; TG, triglyceride; LDL-C, low-density lipoprotein cholesterol; HDL-C, high-density lipoprotein cholesterol. 95%CI did not contain 1 referred to *P*<0.05.)

| Supplementary Table 1. Significant multiple group comparisons of baseline characteristic of included population. | | | | | |
| --- | --- | --- | --- | --- | --- |
| Characteristics | Actual weight: Non-Overweight/ Obesity | | Actual weight: Overweight/ Obesity | | Significant multiple group comparisons† |
|  | Group 1: Perceived Non-Overweight/ Obesity (n=9996) | Group 2: Perceived Overweight/ Obesity (n=2035) | Group 3: Perceived Non-Overweight/ Obesity (n=309) | Group 4: Perceived Overweight/ Obesity (n=1739) |  |
| Age, year, Mean (SD) | 10.9 (3.3) | 12.4 (3.0) | 9.2 (2.9) | 11.4 (3.3) | Group 2 > 4 > 3 |
| BMI values, Mean (SD) |  |  |  |  |  |
| 5-9 years old | 15.6 (1.6) | 18.1 (1.8) | 21.1 (3.4) | 22.6 (2.9) | Group 4 > 3 > 2 >1 |
| 10-14 years old | 17.6 (2.0) | 20.4 (1.8) | 24.4 (3.2) | 26.0 (2.9) | Group 4 > 3 > 2 >1 |
| 15-19 years old | 19.3 (1.9) | 21.9 (2.0) | 26.5 (2.4) | 28.1 (3.1) | Group 4 > 3 > 2 >1 |
| *Sex, n (%)* |  |  |  |  |  |
| Boys | 5135 (51.37) | 892 (43.83) | 134 (43.37) | 898 (51.64) | Group 1 > 2, Group 4 > 2, Group 4 > 3 |
| Girls | 4861 (48.63) | 1143 (56.17) | 175 (56.63) | 841 (48.36) | Group 2 > 1, Group 2 > 4, Group 3 > 1, Group 3 > 4 |
| *Residence area, n (%)* |  |  |  |  |  |
| Urban | 5154 (51.6) | 1147 (56.4) | 157 (50.8) | 1013 (58.3) | Group 4 > 3 |
| Rural | 4842 (48.4) | 888 (43.6) | 152 (49.2) | 726 (41.8) | Group 3 > 4 |
| *Ethnicity, n (%)* |  |  |  |  |  |
| Han | 9248 (92.5) | 1875 (92.1) | 295 (95.5) | 1623 (93.3) | NS |
| Hui | 354 (3.5) | 83 (4.1) | 5 (1.6) | 41 (2.4) | Group 1 > 3, Group 2 > 3 |
| Tibetan | 20 (0.2) | 8 (0.4) | 0 (0.0) | 3 (0.2) | NS |
| Mongolian | 145 (1.5) | 17 (0.8) | 0 (0.0) | 21 (1.2) | Group 1 > 3 |
| Other | 229 (2.3) | 52 (2.6) | 9 (2.9) | 51 (2.9) | NS |
| *Paternal weight status, n (%)* |  |  |  |  |  |
| Normal | 6062 (60.6) | 1108 (54.5) | 133 (43.0) | 703 (40.4) | Group 1 > 3, Group 1 > 4 |
| Overweight | 3122 (31.2) | 679 (33.4) | 119 (38.5) | 697 (40.1) | Group 4 > 2 > 1 |
| Obesity | 812 (8.1) | 248 (12.2) | 57 (18.5) | 339 (19.5) | Group 4 > 2 > 1 |
| *Maternal weight status, n (%)* |  |  |  |  |  |
| Normal | 8246 (82.5) | 1540 (75.7) | 220 (71.2) | 1166 (67.1) | Group 1 > 3, Group 1 > 4 |
| Overweight | 1462 (14.6) | 403 (19.8) | 72 (23.3) | 427 (24.6) | Group 4 > 2 > 1 |
| Obesity | 288 (2.9) | 92 (4.5) | 17 (5.5) | 146 (8.4) | Group 4 > 2 > 1 |
| *Paternal educational attainment, n (%)* |  |  |  |  |  |
| Primary school or below | 665 (6.7) | 126 (6.2) | 18 (5.8) | 96 (5.5) | NS |
| Secondary or equivalent | 5838 (58.4) | 1183 (58.1) | 189 (61.2) | 1046 (60.2) | NS |
| Junior college or above | 3493 (34.9) | 726 (35.7) | 102 (33.0) | 597 (34.3) | NS |
| *Maternal educational attainment, n (%)* |  |  |  |  |  |
| Primary school or below | 884 (8.8) | 175 (8.6) | 28 (9.1) | 133 (7.7) | NS |
| Secondary or equivalent | 5740 (57.4) | 1148 (56.4) | 188 (60.8) | 1016 (58.4) | NS |
| Junior college or above | 3372 (33.7) | 712 (35.0) | 93 (30.1) | 590 (33.9) | NS |
| *Monthly household income, n (%)* |  |  |  |  |  |
| < 5000 CNY | 8425 (84.3) | 1729 (85.0) | 252 (81.6) | 1428 (82.1) | Group 2 > 3 |
| ≥50000 CNY | 1571 (15.7) | 306 (15.0) | 57 (18.5) | 311 (17.9) | NS |
| *Abbreviation: BMI, body mass index; CNY, Chinese yuan.  †One-way ANOVA and Chi-squared test with Holm Bonferroni correction was used to account for multiple comparisons. NS, not significant. | | | | | |

| Supplementary Table 2. Consistency analysis of perception of child's weight and actual weight status. | | | | |
| --- | --- | --- | --- | --- |
| Population | Perception of weight | Actual weight status | | Kappa† |
|  |  | Non-overweight/ obesity, n (%) | Overweight/ Obesity, n (%) |  |
| **Total population (n=14079)** | Non-overweight/ obesity | **9996 (70.8)** | 309 (2.2) | 0.504 |
|  | Overweight/ obesity | 2035 (14.5) | **1739 (12.4)** |  |
| Boys (n=7059) | Non-overweight/ obesity | **5135 (72.7)** | 134 (1.9) | 0.554 |
|  | Overweight/ obesity | 892 (12.6) | **898 (12.7)** |  |
| Girls (n=7020) | Non-overweight/ obesity | **4861 (69.2)** | 175 (2.5) | 0.457 |
|  | Overweight/ obesity | 1143 (16.3) | **841 (11.98)** |  |
| 5-9 years old (n=5380) | Non-overweight/ obesity | **4161 (77.3)** | 194 (3.6) | 0.600 |
|  | Overweight/ obesity | 415 (7.7) | **610 (11.3)** |  |
| 10-14 years old (n=5494) | Non-overweight/ obesity | **3765 (68.5)** | 92 (1.7) | 0.472 |
|  | Overweight/ obesity | 941 (17.1) | **696 (12.7)** |  |
| 15-19 years old (n=3205) | Non-overweight/ obesity | **2070 (64.6)** | 23 (0.7) | 0.439 |
|  | Overweight/ obesity | 679 (21.2) | **433 (13.5)** |  |
| Rural area (n=6608) | Non-overweight/ obesity | **4842 (73.3)** | 152 (2.3) | 0.496 |
|  | Overweight/ obesity | 888 (13.4) | **726 (11.0)** |  |
| Urban area (n=7471) | Non-overweight/ obesity | **5154 (69.0)** | 157 (2.1) | 0.509 |
|  | Overweight/ obesity | 1147 (15.4) | **1013 (13.6)** |  |
| *The rates of children’s self-perceptions about their body weight which was in accordance with their actual BMI status are presented in bold. The rates for children’s underestimation are presented in the light grey colored cells. The rates for children’s overestimation are presented in the deep grey colored cells. | | | | |
| †Agreements between the perceived weight and actual weight status in children and adolescents were evaluated using Kappa test. | | | | |

| Supplementary Table 3. Prevalence of abnormal cardio-metabolic markers by self-perception combined with actual weight status in sub-groups. | | | | | | |
| --- | --- | --- | --- | --- | --- | --- |
| Cardio-metabolic markers, n (%) | Actual weight: Non-Overweight/ Obesity | | Actual weight: Overweight/ Obesity | | *P*-value | Significant multiple group comparisons* |
|  | Group 1: Perceived Non-Overweight/ Obesity | Group 2: Perceived Overweight/ Obesity | Group 3: Perceived Non-Overweight/ Obesity | Group 4: Perceived Overweight/ Obesity |  |  |
| ***5-9 years old*** | (n=4161) | (n=415) | (n=194) | (n=610) |  |  |
| Abdominal obesity | 27 (0.6) | 28 (6.7) | 73 (37.6) | 425 (69.7) | <0.01 | Group 4 >3 > 2 > 1 |
| Elevated BP | 664 (16.0) | 99 (23.9) | 71 (36.6) | 227 (37.2) | <0.01 | Group 4 > 2 > 1, Group 3 > 2 > 1 |
| High blood glucose | 46 (1.1) | 2 (0.5) | 5 (2.6) | 14 (2.3) | 0.039 | Group 3 >2 |
| Dyslipidemia | 922 (22.2) | 127 (30.6) | 75 (38.7) | 286 (46.9) | <0.01 | Group 4 > 2 > 1, Group 3 > 1 |
| *High TC* | 249 (6.0) | 33 (8.0) | 14 (7.2) | 61 (10.0) | 0.001 | Group 4 >1 |
| *High TG* | 617 (14.8) | 83 (20.0) | 56 (28.9) | 223 (36.6) | <0.01 | Group 4 >3 > 2 > 1 |
| *High LDL-C* | 129 (3.1) | 18 (4.3) | 8 (4.1) | 39 (6.4) | 0.001 | Group 4 >1 |
| *Low HDL-C* | 289 (6.9) | 45 (10.8) | 25 (12.9) | 113 (18.5) | <0.01 | Group 4 > 2 > 1, Group 3 > 1 |
| ***10-14 years old*** | (n=3765) | (n=941) | (n=92) | (n=696) |  |  |
| Abdominal obesity | 20 (0.5) | 43 (4.6) | 34 (37.0) | 462 (66.4) | <0.01 | Group 4 >3 > 2 > 1 |
| Elevated BP | 563 (15.0) | 191 (20.3) | 34 (37.0) | 290 (41.7) | <0.01 | Group 4 > 2 > 1, Group 3 > 2 > 1 |
| High blood glucose | 63 (1.7) | 12 (1.3) | 3 (3.3) | 24 (3.4) | 0.061 | NS |
| Dyslipidemia | 1143 (30.4) | 357 (37.9) | 44 (47.8) | 358 (51.4) | <0.01 | Group 4 > 2 > 1, Group 3 > 1 |
| *High TC* | 155 (4.1) | 47 (5.0) | 5 (5.4) | 57 (8.2) | 0.001 | Group 4 >1, Group 4 >2 |
| *High TG* | 785 (20.8) | 253 (26.9) | 33 (35.9) | 266 (38.2) | <0.01 | Group 4 > 2 > 1, Group 3 > 1 |
| *High LDL-C* | 82 (2.2) | 31 (3.3) | 5 (5.4) | 38 (5.5) | <0.01 | Group 4 >1 |
| *Low HDL-C* | 414 (11.0) | 169 (18.0) | 19 (20.7) | 189 (27.2) | <0.01 | Group 4 > 2 > 1 |
| ***15-19 years old*** | (n=2070) | (n=679) | (n=23) | (n=433) |  |  |
| Abdominal obesity | 10 (0.5) | 32 (4.7) | 11 (47.8) | 274 (63.3) | <0.01 | Group 4 >3 > 2 > 1 |
| Elevated BP | 347 (16.8) | 135 (19.9) | 10 (43.5) | 197 (45.5) | <0.01 | Group 4 > 2 > 1, Group 3 > 2 > 1 |
| High blood glucose | 60 (2.9) | 16 (2.4) | 1 (4.3) | 20 (4.6) | 0.746 | NS |
| Dyslipidemia | 514 (24.8) | 209 (30.8) | 9 (39.1) | 219 (50.6) | <0.01 | Group 4 > 2 > 1 |
| *High TC* | 82 (4.0) | 33 (4.9) | 1 (4.3) | 30 (6.9) | 0.087 | NS |
| *High TG* | 286 (13.8) | 112 (16.5) | 5 (21.7) | 142 (32.8) | <0.01 | Group 4 > 2, Group 4 > 1 |
| *High LDL-C* | 32 (1.5) | 22 (3.2) | 1 (4.3) | 23 (5.3) | <0.01 | Group 4 > 1 |
| *Low HDL-C* | 288 (13.9) | 109 (16.1) | 6 (26.1) | 138 (31.9) | <0.01 | Group 4 > 2, Group 4 > 1 |
| ***Rural area*** | (n=4842) | (n=888) | (n=152) | (n=726) |  |  |
| Abdominal obesity | 20 (0.4) | 43 (4.8) | 59 (38.8) | 555 (76.4) | <0.01 | Group 4 >3 > 2 > 1 |
| Elevated BP | 852 (17.6) | 193 (21.7) | 67 (44.1) | 383 (52.8) | <0.01 | Group 4 >2, Group 4 >1, Group 3 >2, Group 3 >1 |
| High blood glucose | 101 (2.1) | 23 (2.6) | 6 (3.9) | 32 (4.4) | 0.118 | NS |
| Dyslipidemia | 752 (15.5) | 225 (25.3) | 54 (35.5) | 383 (52.8) | <0.01 | Group 4 > 2 > 1, Group 3 > 2 > 1 |
| *High TC* | 135 (2.8) | 43 (4.8) | 10 (6.6) | 59 (8.1) | <0.01 | Group 4 >1 |
| *High TG* | 328 (6.8) | 118 (13.3) | 36 (23.7) | 255 (35.1) | <0.01 | Group 4 > 2 > 1, Group 3 > 2 > 1 |
| *High LDL-C* | 57 (1.2) | 22 (2.5) | 5 (3.3) | 36 (5.0) | <0.01 | Group 4 >1, Group 4 >2 |
| *Low HDL-C* | 372 (7.7) | 113 (12.7) | 24 (15.8) | 216 (29.8) | <0.01 | Group 4 > 3 > 1, Group 4 > 2 > 1 |
| ***Urban area*** | (n=5154) | (n=1147) | (n=157) | (n=1013) |  |  |
| Abdominal obesity | 37 (0.7) | 60 (5.2) | 59 (37.6) | 608 (60.0) | <0.01 | Group 4 >3 > 2 > 1 |
| Elevated BP | 722 (14.0) | 232 (20.2) | 45 (28.7) | 356 (35.1) | <0.01 | Group 4 > 2 > 1, Group 3 > 2 > 1 |
| High blood glucose | 68 (1.3) | 9 (0.8) | 3 (1.9) | 21 (2.1) | 0.083 | NS |
| Dyslipidemia | 1941 (37.7) | 479 (41.8) | 69 (43.9) | 490 (48.4) | <0.01 | Group 4 > 2 > 1 |
| *High TC* | 351 (6.8) | 70 (6.1) | 10 (6.4) | 89 (8.8) | 0.002 | Group 4 > 2 |
| *High TG* | 1360 (26.4) | 330 (28.8) | 53 (33.8) | 367 (36.2) | <0.01 | Group 4 > 2 > 1, Group 3 >1 |
| *High LDL-C* | 186 (3.6) | 51 (4.4) | 7 (4.5) | 64 (6.3) | <0.01 | Group 4 > 2, Group 4 > 1 |
| *Low HDL-C* | 619 (12.0) | 210 (18.3) | 28 (17.8) | 224 (22.1) | <0.01 | Group 4 > 2 > 1, Group 4 > 3 |
| * Chi-squared test with Holm Bonferroni correction was used to account for multiple comparisons. NS, not significant.  BP, blood pressure; TC, total cholesterol; TG, triglyceride; LDL-C, low-density lipoprotein cholesterol; HDL-C, high-density lipoprotein cholesterol. | | | | | | |

| Supplementary Table 4. Children's weight-related attitudes by self-perception combined with actual weight status in sub-groups. | | | | | |
| --- | --- | --- | --- | --- | --- |
| Weight-related attitudes in sub-groups, n (%) | Actual weight: Non-Overweight/ Obesity | | Actual weight: Overweight/ Obesity | | Significant multiple group comparisons* |
|  | Group 1: Perceived Non-Overweight/ Obesity | Group 2: Perceived Overweight/ Obesity | Group 3: Perceived Non-Overweight/ Obesity | Group 4: Perceived Overweight/ Obesity |  |
| **To what extent do you think obesity is bad for health? [greatly or rather greatly]** |  |  |  |  |  |
| 5-9 years old | 3376 (81.1) | 353 (85.1) | 163 (84.0) | 525 (86.1) | Group 4 > 1 |
| 10-14 years old | 2773 (73.7) | 717 (76.2) | 78 (84.8) | 558 (80.2) | Group 3 > 1 |
| 15-19 years old | 1587 (76.7) | 536 (78.9) | 21 (91.3) | 350 (80.8) | NS |
| Rural area | 3251 (67.1) | 695 (78.3) | 117 (77.0) | 647 (89.1) | Group 4 > 1 |
| Urban area | 4485 (87.0) | 911 (79.4) | 145 (92.4) | 786 (77.6) | NS |
| **Are you satisfied with your weight status? [no or rather no]** |  |  |  |  |  |
| 5-9 years old | 1014 (24.4) | 225 (54.2) | 41 (21.1) | 453 (74.3) | Group 4 > 2 > 1, Group 4 > 2 > 3 |
| 10-14 years old | 1134 (30.1) | 670 (71.2) | 45 (48.9) | 637 (91.5) | Group 4 > 2 > 3 > 1 |
| 15-19 years old | 802 (38.7) | 523 (77.0) | 12 (52.2) | 408 (94.2) | Group 4 > 2 > 3, Group 4 > 2 > 1 |
| Rural area | 1253 (25.9) | 615 (69.3) | 40 (26.3) | 685 (94.4) | Group 4 > 2 > 3, Group 4 > 2 > 1 |
| Urban area | 1697 (32.9) | 803 (70.0) | 58 (36.9) | 813 (80.3) | Group 4 > 2 > 3, Group 4 > 2 > 1 |
| **Do you want to change your present weight status? [yes or rather yes]** |  |  |  |  |  |
| 5-9 years old | 1492 (35.9) | 316 (76.1) | 95 (49.0) | 534 (87.5) | Group 4 > 2 > 3 > 1 |
| 10-14 years old | 1593 (42.3) | 772 (82.0) | 71 (77.2) | 664 (95.4) | Group 4 > 2 > 3 > 1 |
| 15-19 years old | 1096 (52.9) | 588 (86.6) | 14 (60.9) | 408 (94.2) | Group 4 > 3, Group 4 > 1, Group 2 > 1 |
| Rural area | 1773 (36.6) | 710 (80.0) | 72 (47.4) | 732 (100.8) | Group 4 > 3, Group 4 > 1, Group 2 > 1 |
| Urban area | 2408 (46.7) | 966 (84.2) | 108 (68.8) | 874 (86.3) | Group 4 > 3 > 1, Group 2 > 3 > 1 |
| **Do you believe you can achieve an ideal weight status through effort? [yes or rather yes]** |  |  |  |  |  |
| 5-9 years old | 2758 (66.3) | 300 (72.3) | 140 (72.2) | 451 (73.9) | Group 4 > 1 |
| 10-14 years old | 2502 (66.5) | 626 (66.5) | 75 (81.5) | 499 (71.7) | NS |
| 15-19 years old | 1348 (65.1) | 439 (64.7) | 19 (82.6) | 305 (70.4) | NS |
| Rural area | 2750 (56.8) | 577 (65.0) | 99 (65.1) | 573 (78.9) | Group 4 > 1 |
| Urban area | 3858 (74.9) | 788 (68.7) | 135 (86.0) | 682 (67.3) | NS |
| * Chi-squared test with Holm Bonferroni correction was used to account for multiple comparisons. NS, not significant. | | | | | |

| Supplementary Table 5. Multivariate odds ratios (OR) and 95% confidence intervals (CI) for abnormal cardio-metabolic markers by groups of self-perception combined with actual weight status, stratified by sex. | | | | |
| --- | --- | --- | --- | --- |
| Cardio-metabolic markers† | Actual weight: Non-Overweight/ Obesity | | Actual weight: Overweight/ Obesity | |
|  | Group 1: Perceived Non-Overweight/ Obesity | Group 2: Perceived Overweight/ Obesity | Group 3: Perceived Non-Overweight/ Obesity | Group 4: Perceived Overweight/ Obesity |
| ***Total population*** | (n=9996) | (n=2035) | (n=309) | (n=1739) |
| Abdominal obesity | 1 (Reference) | 10.49 (7.45-14.76)** | 95.69 (66.69-137.28)** | 355.83 (265.21-477.42)** |
| Elevated BP | 1 (Reference) | 1.30 (1.12-1.51)** | 2.11 (1.51-2.94)** | 2.42 (2.06-2.84)** |
| High blood glucose | 1 (Reference) | 0.93 (0.64-1.36) | 2.24 (1.12-4.46)** | 1.45 (1.03-2.04)* |
| Dyslipidemia | 1 (Reference) | 1.43 (1.29-1.58)** | 1.92 (1.52-2.43)** | 2.68 (2.41-2.99)** |
| *High TC* | 1 (Reference) | 1.31 (1.06-1.63)* | 1.21 (0.76-1.93) | 1.87 (1.53-2.29)** |
| *High TG* | 1 (Reference) | 1.38 (1.23-1.56)** | 2.21 (1.72-2.85)** | 2.77 (2.47-3.11)** |
| *High LDL-C* | 1 (Reference) | 1.64 (1.25-2.16)** | 1.42 (0.78-2.58) | 2.52 (1.97-3.23)** |
| *Low HDL-C* | 1 (Reference) | 1.58 (1.38-1.82)** | 2.06 (1.50-2.83)** | 2.93 (2.57-3.35)** |
| ***Boys*** | (n=5135) | (n=892) | (n=134) | (n=897) |
| Abdominal obesity | 1 (Reference) | 11.79 (6.63-20.97)** | 172.87 (96.58-309.43)** | 564.17 (346.21-919.33)** |
| Elevated BP | 1 (Reference) | 1.45 (1.24-1.69)** | 2.46 (1.69-3.59)** | 2.85 (2.44-3.33)** |
| High blood glucose | 1 (Reference) | 0.92 (0.57-1.47) | 2.26 (0.97-5.29) | 1.38 (0.91-2.08) |
| Dyslipidemia | 1 (Reference) | 1.62 (1.39-1.89)** | 1.92 (1.34-2.75)** | 3.13 (2.69-3.63)** |
| *High TC* | 1 (Reference) | 1.79 (1.31-2.43)** | 2.19 (1.23-3.92)** | 2.20 (1.65-2.93)** |
| *High TG* | 1 (Reference) | 1.35 (1.12-1.62)** | 2.11 (1.41-3.16)** | 3.16 (2.69-3.71)** |
| *High LDL-C* | 1 (Reference) | 2.32 (1.57-3.43)** | 1.97 (0.84-4.61) | 3.16 (2.22-4.51)** |
| *Low HDL-C* | 1 (Reference) | 1.77 (1.45-2.15)** | 1.94 (1.17-3.22)** | 3.05 (2.55-3.66)** |
| ***Girls*** | (n=4861) | (n=1143) | (n=175) | (n=841) |
| Abdominal obesity | 1 (Reference) | 9.55 (6.22-14.66)** | 61.17 (38.39-97.48)** | 250.85 (172.80-364.16)** |
| Elevated BP | 1 (Reference) | 1.30 (1.12-1.51)** | 2.11 (1.51-2.94)** | 2.42 (2.06-2.84)** |
| High blood glucose | 1 (Reference) | 0.95 (0.50-1.78) | 2.28 (0.69-7.50) | 1.65 (0.88-3.07) |
| Dyslipidemia | 1 (Reference) | 1.33 (1.16-1.54)** | 1.93 (1.42-2.62)** | 2.32 (1.99-2.71)** |
| *High TC* | 1 (Reference) | 0.96 (0.71-1.29) | 0.59 (0.26-1.34) | 1.62 (1.23-2.15)** |
| *High TG* | 1 (Reference) | 1.42 (1.21-1.66)** | 2.27 (1.63-3.14)** | 2.42 (2.05-2.85)** |
| *High LDL-C* | 1 (Reference) | 1.19 (0.81-1.75) | 1.12 (0.48-2.58) | 2.04 (1.44-2.90)** |
| *Low HDL-C* | 1 (Reference) | 1.61 (1.32-1.97)** | 2.11 (1.41-3.18)** | 2.95 (2.43-3.59)** |
| **P* < 0.05, ** *P* < 0.01. | | | | |
| †Model: adjusted for age, residence area, ethnicity, incomes, parental educational attainment, parental weight.  BP, blood pressure; TC, total cholesterol; TG, triglyceride; LDL-C, low-density lipoprotein cholesterol; HDL-C, high-density lipoprotein cholesterol. | | | | |

| Supplementary Table 6. Multivariate odds ratios (OR) and 95% confidence intervals (CI) for abnormal cardio-metabolic markers by groups of self-perception combined with actual weight status, stratified by age. | | | | |
| --- | --- | --- | --- | --- |
| Cardio-metabolic markers† | Actual weight: Non-Overweight/ Obesity | | Actual weight: Overweight/ Obesity | |
|  | Group 1: Perceived Non-Overweight/ Obesity | Group 2: Perceived Overweight/ Obesity | Group 3: Perceived Non-Overweight/ Obesity | Group 4: Perceived Overweight/ Obesity |
| ***5-9 years old*** | (n=4161) | (n=415) | (n=194) | (n=610) |
| Abdominal obesity | 1 (Reference) | 10.75 (6.25-18.49)** | 87.23 (53.91-141.13)** | 352.03 (230.71-537.15)** |
| Elevated BP | 1 (Reference) | 2.07 (1.55-2.75)** | 2.13 (1.45-3.13)** | 2.84 (2.26-3.57)** |
| High blood glucose | 1 (Reference) | 0.42 (0.10-1.76) | 2.30 (0.89-5.92) | 1.42 (0.68-2.99) |
| Dyslipidemia | 1 (Reference) | 1.53 (1.23-1.91)** | 2.08 (1.54-2.80)** | 2.92 (2.44-3.50)** |
| *High TC* | 1 (Reference) | 1.35 (0.92-1.98) | 1.26 (0.72-2.21) | 1.73 (1.28-2.35)** |
| *High TG* | 1 (Reference) | 1.46 (1.12-1.89)** | 2.36 (1.70-3.28)** | 3.40 (2.80-4.12)** |
| *High LDL-C* | 1 (Reference) | 1.55 (0.95-2.52) | 1.19 (0.54-2.59) | 2.12 (1.45-3.11)** |
| *Low HDL-C* | 1 (Reference) | 1.77 (1.27-2.47)** | 1.87 (1.19-2.92)** | 3.21 (2.51-4.11)** |
| ***10-14 years old*** | (n=3765) | (n=941) | (n=92) | (n=696) |
| Abdominal obesity | 1 (Reference) | 10.24 (5.89-17.81)** | 97.00 (51.00-184.51)** | 356.32 (217.62-583.42)** |
| Elevated BP | 1 (Reference) | 1.34 (1.13-1.58)** | 2.89 (1.89-4.42)** | 3.01 (2.53-3.59)** |
| High blood glucose | 1 (Reference) | 0.91 (0.50-1.66) | 2.43 (0.74-8.00) | 1.83 (1.08-3.09)** |
| Dyslipidemia | 1 (Reference) | 1.42 (1.22-1.65)** | 1.70 (1.11-2.59)** | 2.33 (1.97-2.77)** |
| *High TC* | 1 (Reference) | 1.30 (0.93-1.80) | 1.34 (0.53-3.35) | 1.97 (1.42-2.74)** |
| *High TG* | 1 (Reference) | 1.40 (1.19-1.65)** | 2.15 (1.38-3.33)** | 2.21 (1.85-2.65)** |
| *High LDL-C* | 1 (Reference) | 1.53 (1.00-2.32)* | 2.49 (0.98-6.33) | 2.43 (1.62-3.67)** |
| *Low HDL-C* | 1 (Reference) | 1.82 (1.50-2.21)** | 2.11 (1.26-3.55)** | 2.96 (2.42-3.62)** |
| ***15-19 years old*** | (n=2070) | (n=679) | (n=23) | (n=433) |
| Abdominal obesity | 1 (Reference) | 12.15 (5.25-28.12)** | 255.86 (83.79-781.24)** | 435.01 (201.11-940.92)** |
| Elevated BP | 1 (Reference) | 1.26 (1.04-1.53)* | 2.07 (0.90-4.76) | 2.55 (2.05-3.17)** |
| High blood glucose | 1 (Reference) | 1.10 (0.64-1.88) | 1.56 (0.20-12.19) | 1.18 (0.66-2.10) |
| Dyslipidemia | 1 (Reference) | 1.50 (1.23-1.82)** | 1.62 (0.65-4.00) | 3.24 (2.59-4.04)** |
| *High TC* | 1 (Reference) | 1.04 (0.68-1.59) | 1.49 (0.20-11.34) | 1.80 (1.15-2.82)** |
| *High TG* | 1 (Reference) | 1.32 (1.04-1.69)** | 1.18 (0.34-4.03) | 3.38 (2.64-4.34)** |
| *High LDL-C* | 1 (Reference) | 1.85 (1.05-3.26)** | -‡ | 3.61 (2.06-6.30)** |
| *Low HDL-C* | 1 (Reference) | 1.46 (1.14-1.87)** | 2.70 (1.03-7.09)** | 2.97 (2.31-3.82)** |
| **P* < 0.05, ** *P* < 0.01. | | | | |
| †Model: adjusted for sex, age, residence area, ethnicity, incomes, parental educational attainment, parental weight.  BP, blood pressure; TC, total cholesterol; TG, triglyceride; LDL-C, low-density lipoprotein cholesterol; HDL-C, high-density lipoprotein cholesterol.  ‡Data were not shown if the number of participants in this group was too small to analyze. | | | | |

| Supplementary Table 7. Multivariate odds ratios (OR) and 95% confidence intervals (CI) for abnormal cardio-metabolic markers by groups of self-perception combined with actual weight status, stratified by residence areas. | | | | |
| --- | --- | --- | --- | --- |
| Cardio-metabolic markers† | Actual weight: Non-Overweight/ Obesity | | Actual weight: Overweight/ Obesity | |
|  | Group 1: Perceived Non-Overweight/ Obesity | Group 2: Perceived Overweight/ Obesity | Group 3: Perceived Non-Overweight/ Obesity | Group 4: Perceived Overweight/ Obesity |
| ***Rural area*** | (n=4842) | (n=888) | (n=152) | (n=726) |
| Abdominal obesity | 1 (Reference) | 10.81 (6.36-18.39)** | 110.20 (64.21-189.13)** | 440.77 (280.20-693.36)** |
| Elevated BP | 1 (Reference) | 1.33 (1.12-1.57)** | 1.68 (1.13-2.50)** | 2.81 (2.36-3.34)** |
| High blood glucose | 1 (Reference) | 1.04 (0.59-1.82) | 2.62 (1.11-6.20)** | 1.45 (0.85-2.46) |
| Dyslipidemia | 1 (Reference) | 1.47 (1.25-1.72)** | 2.78 (1.99-3.87)** | 3.09 (2.62-3.65)** |
| *High TC* | 1 (Reference) | 1.44 (0.98-2.12) | 1.95 (1.03-3.70)* | 2.62 (1.87-3.66)** |
| *High TG* | 1 (Reference) | 1.49 (1.23-1.79)** | 2.67 (1.84-3.88)** | 3.17 (2.64-3.81)** |
| *High LDL-C* | 1 (Reference) | 1.56 (0.91-2.66) | 1.95 (0.77-4.93) | 3.31 (2.13-5.14)** |
| *Low HDL-C* | 1 (Reference) | 1.56 (1.27-1.91)** | 2.93 (1.97-4.38)** | 2.95 (2.42-3.60)** |
| ***Urban area*** | (n=5154) | (n=1147) | (n=157) | (n=1013) |
| Abdominal obesity | 1 (Reference) | 9.95 (6.36-15.55)** | 87.45 (53.64-142.57)** | 304.65 (206.95-448.48)** |
| Elevated BP | 1 (Reference) | 1.37 (1.16-1.60)** | 3.41 (2.40-4.86)** | 2.71 (2.32-3.17)** |
| High blood glucose | 1 (Reference) | 0.88 (0.53-1.47) | 1.77 (0.54-5.78) | 1.50 (0.95-2.36) |
| Dyslipidemia | 1 (Reference) | 1.38 (1.20-1.58)** | 1.37 (0.98-1.90) | 2.44 (2.12-2.81)** |
| *High TC* | 1 (Reference) | 1.25 (0.97-1.61) | 0.82 (0.41-1.63) | 1.57 (1.22-2.01)** |
| *High TG* | 1 (Reference) | 1.27 (1.09-1.49)** | 1.93 (1.36-2.72)** | 2.58 (2.22-2.99)** |
| *High LDL-C* | 1 (Reference) | 1.65 (1.20-2.27)** | 1.19 (0.55-2.59) | 2.23 (1.66-3.01)** |
| *Low HDL-C* | 1 (Reference) | 1.59 (1.32-1.92)** | 1.23 (0.71-2.12) | 2.94 (2.46-3.51)** |
| **P* < 0.05, ** *P* < 0.01. | | | | |
| †Model: adjusted for sex, age, ethnicity, incomes, parental educational attainment, parental weight.  BP, blood pressure; TC, total cholesterol; TG, triglyceride; LDL-C, low-density lipoprotein cholesterol; HDL-C, high-density lipoprotein cholesterol. | | | | |


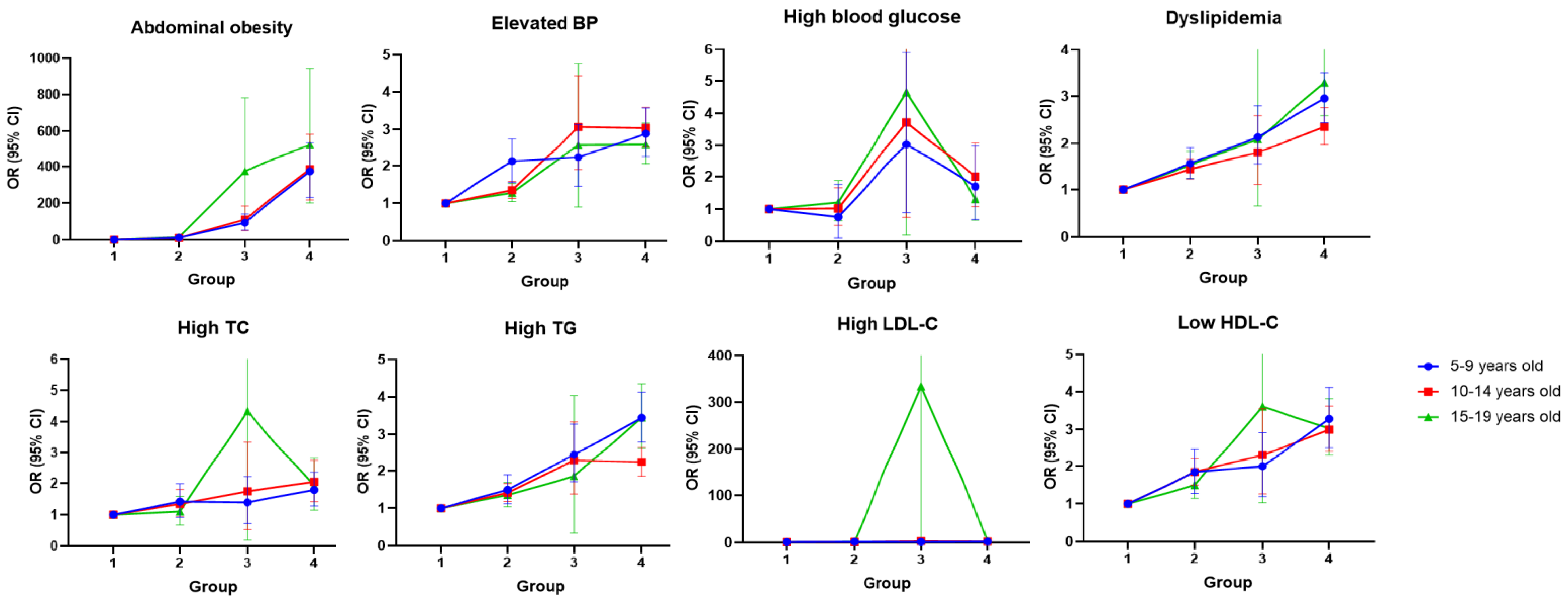


Supplementary Figure 1. Multivariate odds ratios (OR) and 95% confidence intervals (CI) for abnormal cardio-metabolic markers by groups of self-perception combined with actual weight status, stratified by age. (Group 1: non-overweight/obese participants with accurate estimation; Group 2: weight over-estimators; Group 3: weight under-estimators; Group 4: overweight/obese participants with accurate estimation. Group 1 was considered as a reference group; BP, blood pressure; TC, total cholesterol; TG, triglyceride; LDL-C, low-density lipoprotein cholesterol; HDL-C, high-density lipoprotein cholesterol. 95%CI did not contain 1 referred to *P*<0.05.)


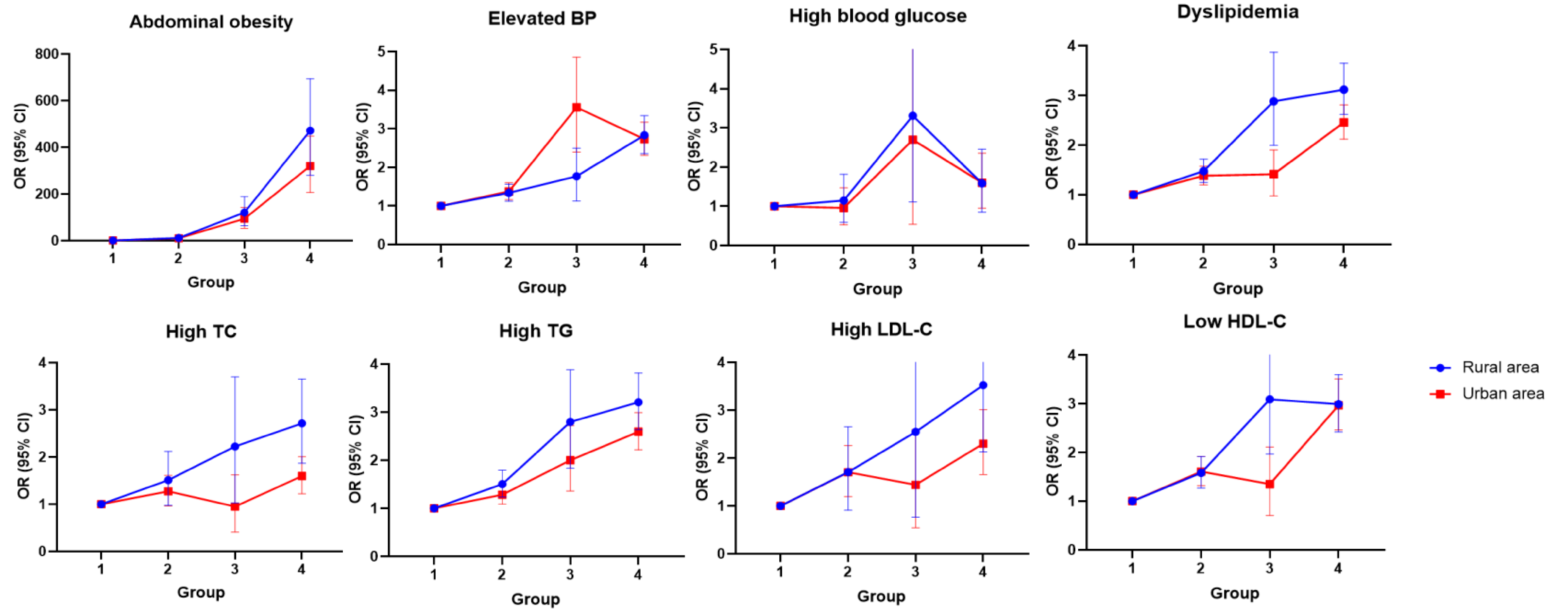


Supplementary Figure 2. Multivariate odds ratios (OR) and 95% confidence intervals (CI) for abnormal cardio-metabolic markers by groups of self-perception combined with actual weight status, stratified by residence areas. (Group 1: non-overweight/obese participants with accurate estimation; Group 2: weight over-estimators; Group 3: weight under-estimators; Group 4: overweight/obese participants with accurate estimation. Group 1 was considered as a reference group; BP, blood pressure; TC, total cholesterol; TG, triglyceride; LDL-C, low-density lipoprotein cholesterol; HDL-C, high-density lipoprotein cholesterol. 95%CI did not contain 1 referred to *P*<0.05.)
